# Supplementary figures and images for: Robust blind spectral unmixing for fluorescence microscopy using unsupervised learning
Source: PLoS One. 2019 Dec 2;14(12):e0225410. doi: 10.1371/journal.pone.0225410 (PMC6886781; doi:10.1371/journal.pone.0225410)

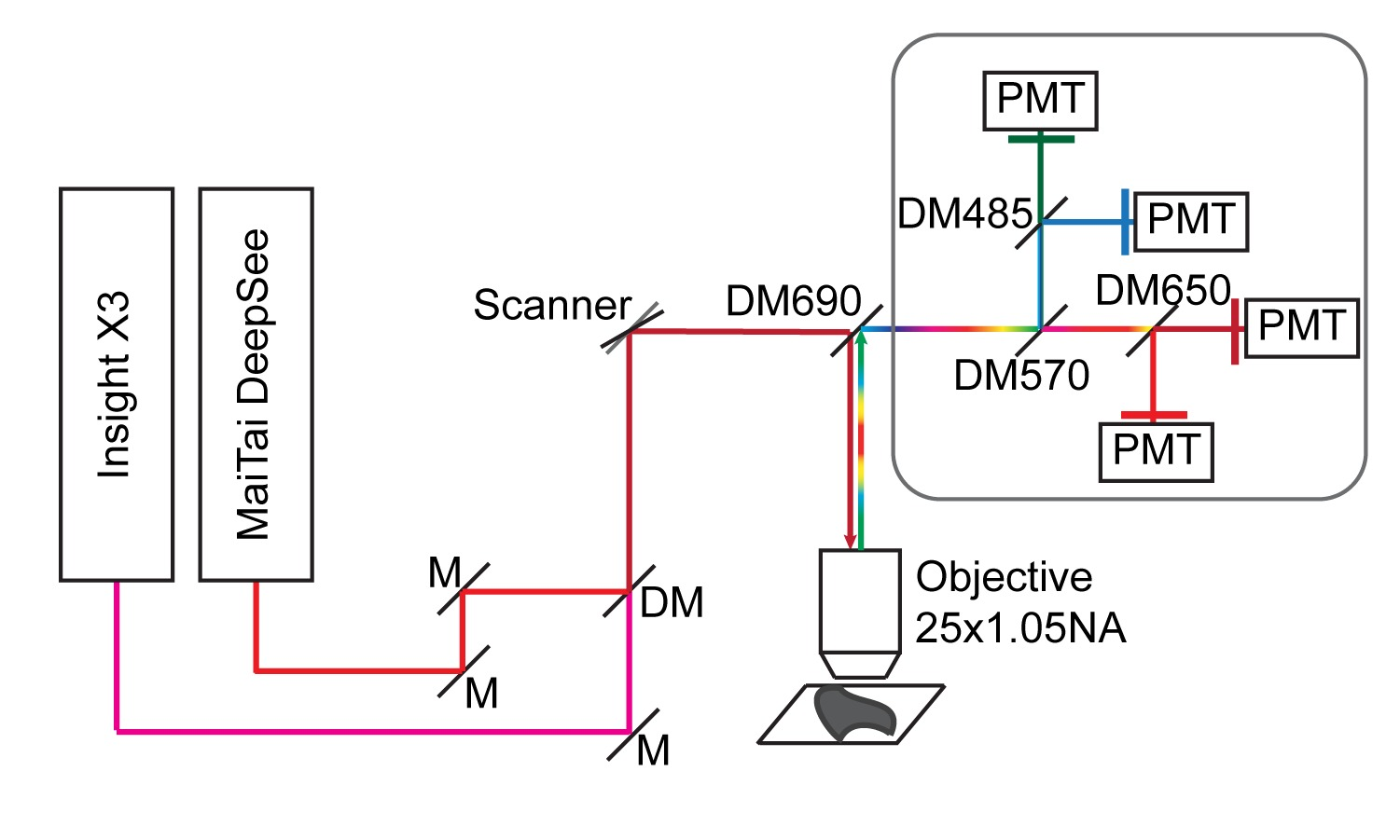

Supplement: S1 Fig — The system (Olympus FVMPE-RS) was equipped with two two-photon lasers and four PMTs. 25× water immersion objective was used. M: mirror, DM: dichroic mirror, Scanner: galvanometer scanner, PMT: photomultiplier tube. The Blue/Green (420-460nm/495-540nm), and Red/fRed (575-630nm/645-685nm) filter cubes setup is shown. (TIF) [file pone.0225410.s001.tif]

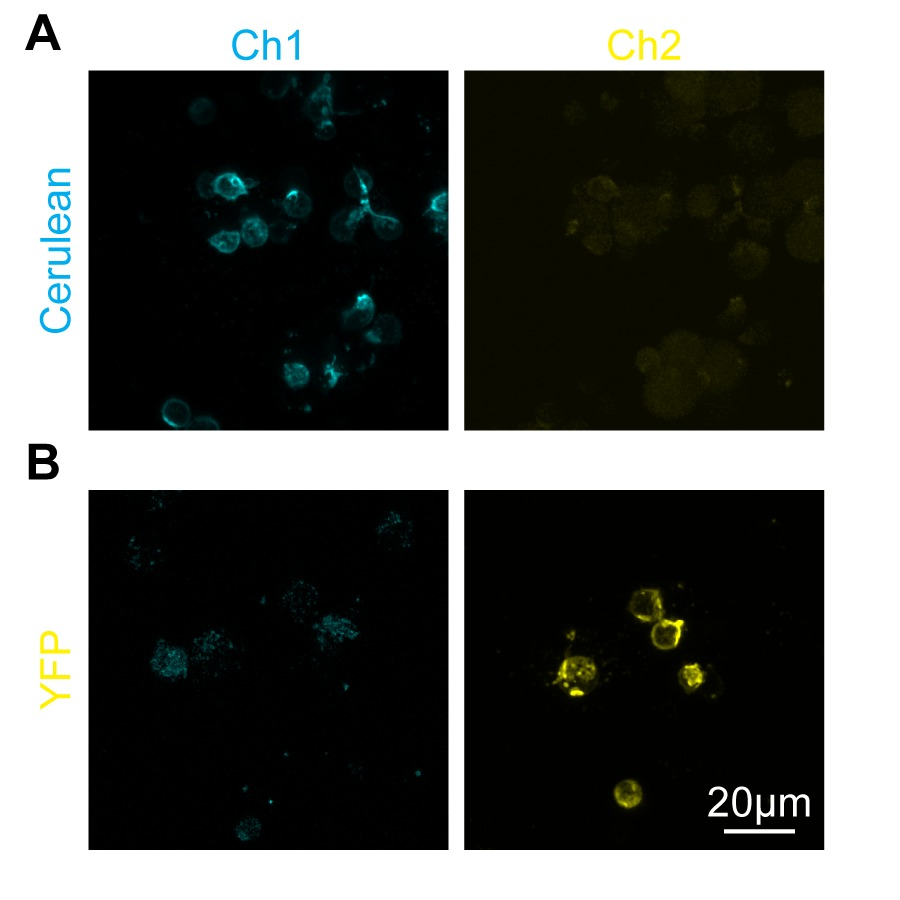

Supplement: S2 Fig — T cells only transduced with Cerulean or YFP virus mixed with APCs and imaged with the same conditions as in Fig 6. (A) Cells transduced with Cerulean expressing virus. Cerulean signals showed only in CFP channel (Ch1). (B) Cells transduced with YFP virus. YFP signals were only detected by the YFP channel (Ch2). There was no cross-talk between CFP and YFP channels. APCs showed weak autofluorescence. (TIF) [file pone.0225410.s002.tif]

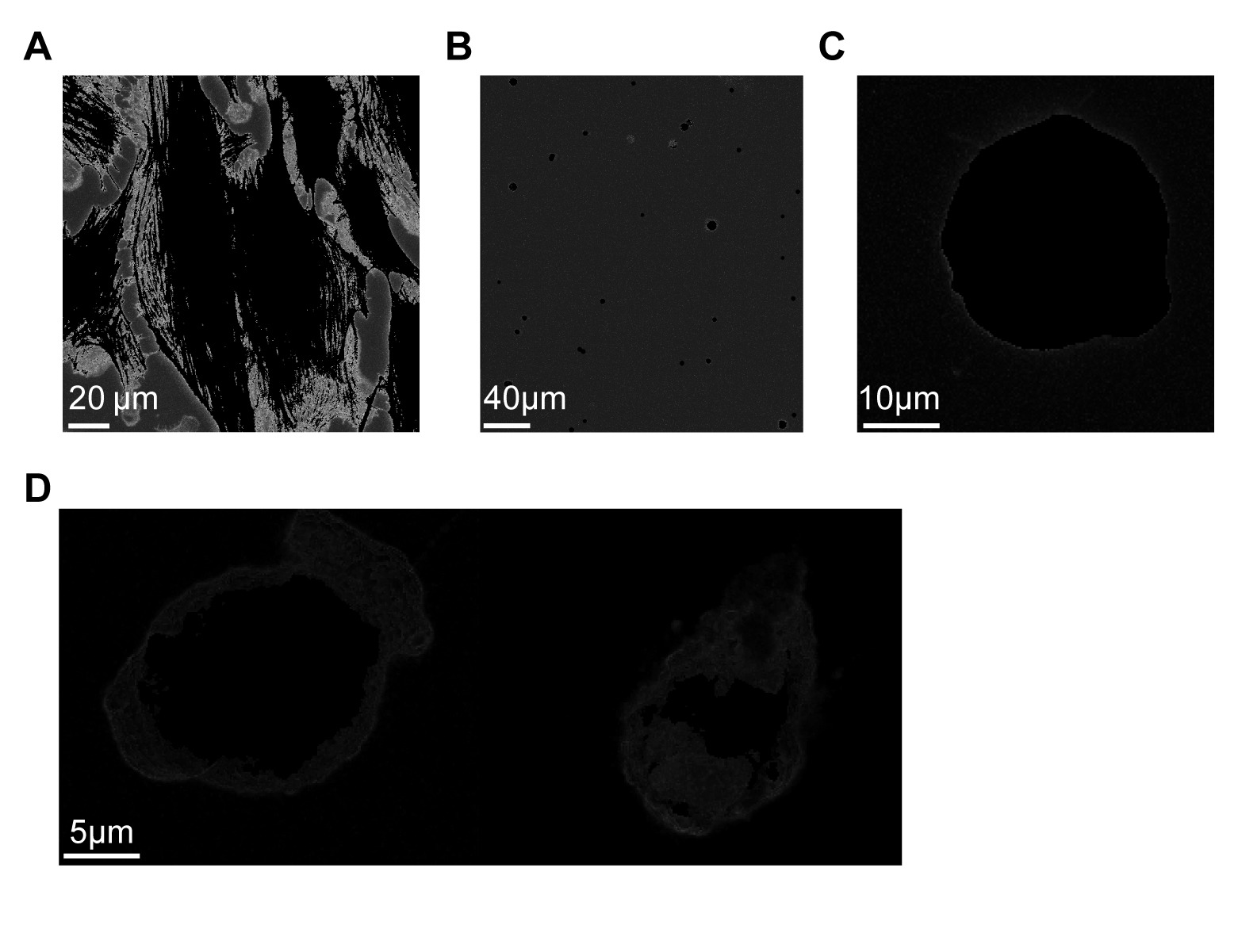

Supplement: S3 Fig — (A) The separated background image by LUMoS of the BPAE cells image in Fig 3. (B) The separated background image by LUMoS of the multi-color beads image in Fig 4. (C) The separated background image by LUMoS of the colorful cell image in Fig 5. (D) The separated background image of the imaged cells in Fig 6. (TIF) [file pone.0225410.s003.tif]

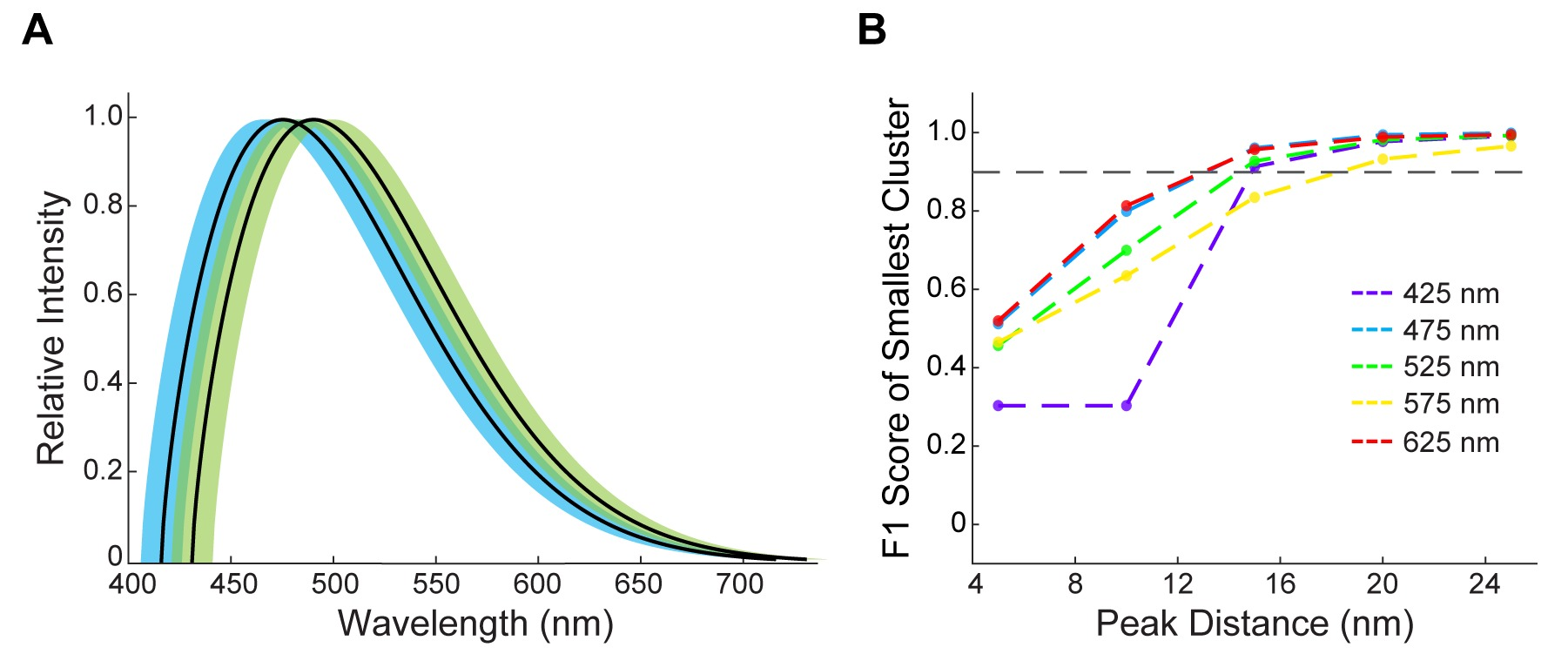

Supplement: S4 Fig — (A) Synthetic emission spectra of two fluorophores with peak emissions at 475 nm and 490 nm. 10 nm standard deviations for each spectra are shown in shaded area. (B) The performance of LUMoS for synthetic images of two fluorophores with variable distances between emission peaks. The cluster size ratio was fixed at 0.2, number of fluorophores at 2 and SNR at 10. For each color plotted, the peak of the lower wavelength fluorophore was fixed while the peak of the higher wavelength fluorophore was varied. Results of 10 simulations were averaged to obtain the final results. (TIF) [file pone.0225410.s004.tif]
